# Central and peripheral markers of neurodegeneration and monocyte activation in HIV-associated neurocognitive disorders

**DOI:** 10.1007/s13365-015-0333-3

**Published:** 2015-03-17

**Authors:** Jennifer L McGuire, Alexander J Gill, Steven D Douglas, Dennis L Kolson, CNS HIV Anti-Retroviral Therapy Effects Research (CHARTER) group

**Affiliations:** 1 Division of Neurology, The Children’s Hospital of Philadelphia, Philadelphia, PA 19104 USA; 2 Division of Allergy and Immunology, The Children’s Hospital of Philadelphia, Philadelphia, PA 19104 USA; 3 Department of Neurology, Perelman School of Medicine at the University of Pennsylvania, Philadelphia, PA 19104 USA; 4 Department of Pediatrics, Perelman School of Medicine at the University of Pennsylvania, Philadelphia, PA 19104 USA; 5 The Children’s Hospital of Philadelphia Research Institute, Philadelphia, PA USA

**Keywords:** Neurofilament, NFL, HIV, HAND, Monocyte activation, Macrophage, Neurodegeneration, Neuroinflammation, sCD163, sCD14, pNFH

## Abstract

HIV-associated neurocognitive disorders (HAND) affect up to 50 % of HIV-infected adults, independently predict HIV morbidity/mortality, and are associated with neuronal damage and monocyte activation. Cerebrospinal fluid (CSF) neurofilament subunits (NFL, pNFH) are sensitive surrogate markers of neuronal damage in several neurodegenerative diseases. In HIV, CSF NFL is elevated in individuals with and without cognitive impairment, suggesting early/persistent neuronal injury during HIV infection. Although individuals with severe cognitive impairment (HIV-associated dementia (HAD)) express higher CSF NFL levels than cognitively normal HIV-infected individuals, the relationships between severity of cognitive impairment, monocyte activation, neurofilament expression, and systemic infection are unclear. We performed a retrospective cross-sectional study of 48 HIV-infected adults with varying levels of cognitive impairment, not receiving antiretroviral therapy (ART), enrolled in the CNS Anti-Retroviral Therapy Effects Research (CHARTER) study. We quantified NFL, pNFH, and monocyte activation markers (sCD14/sCD163) in paired CSF/plasma samples. By examining subjects off ART, these correlations are not confounded by possible effects of ART on inflammation and neurodegeneration. We found that CSF NFL levels were elevated in individuals with HAD compared to cognitively normal or mildly impaired individuals with CD4+ T-lymphocyte nadirs ≤200. In addition, CSF NFL levels were significantly positively correlated to plasma HIV-1 RNA viral load and negatively correlated to plasma CD4+ T-lymphocyte count, suggesting a link between neuronal injury and systemic HIV infection. Finally, CSF NFL was significantly positively correlated with CSF pNFH, sCD163, and sCD14, demonstrating that monocyte activation within the CNS compartment is directly associated with neuronal injury at all stages of HAND.

## Introduction

HIV-associated neurocognitive disorders (HAND) are a common complication of HIV infection in the era of combined antiretroviral therapy (cART) that independently predict overall morbidity and mortality (Ellis et al. 2007; Ellis et al. 1997; Valcour et al. 2011; Vivithanaporn et al. 2010). The clinical sub-syndromes of HAND vary in severity of cognitive impairment and associated functioning and include asymptomatic neurocognitive impairment (ANI), mild neurocognitive disorder (MND), and HIV-associated dementia (HAD) (Antinori et al. 2007). Although HIV does not infect neurons, cognitive impairment in HIV is associated with pathological evidence of neuronal damage (synaptic loss, dendritic simplification), as well as infection and activation of central nervous system (CNS) infiltrating monocyte-derived macrophages (Cherner et al. 2002; Ellis et al. 2007; Masliah et al. 1997). Up to 30 % of HIV-infected individuals qualify for a diagnosis of ANI, 10–30 % for MND, and 2–8 % for HAD (Ellis et al. 2007; McArthur et al. 2010); each diagnosis is based on neurocognitive testing and assessments of daily functioning in the absence of pre-existing or confounding diagnoses that may independently result in cognitive impairment. The temporal progression among these subtypes of HAND is not consistently linear, although they appear to be clinically and pathologically related (Ellis et al. 2007; McArthur et al. 2010; Tan and McArthur 2012). Individuals with ANI have an increased risk of progression to functional decline compared to neurocognitively normal HIV-infected individuals (Ellis et al. 2007; Grant et al. 2014; Heaton et al. 2012; McArthur and Brew 2010; McArthur et al. 2010). In addition, mild, moderate, and severe cognitive impairment has been associated with synaptodendritic injury (Ellis et al. 2007; Masliah et al. 1997; McArthur et al. 2010; Tan and McArthur 2012), although the relationship between pathological abnormalities and severity of cognitive impairment has not been fully defined (Gelman et al. 2012).

Neurofilaments (NFs) are structural proteins specific to neurons that are released into the cerebrospinal fluid (CSF) and blood following axonal disruption or degeneration (Gresle et al. 2011; Julien 1999; Pasol et al. 2010). The neurofilament core chains are of low, medium, or high molecular weight (NFL, NFM, NFH, respectively) with varying degrees of phosphorylation; these proteins are expressed in a stereotypic and phylogenetically conserved manner throughout neuronal development (Mellgren et al. 2007; Szaro and Strong 2010). Elevations in CSF NFL are a sensitive surrogate marker of neuronal damage, as evidenced by pathological white matter changes, in several neurodegenerative diseases, including Alzheimer’s disease, subcortical vascular dementia (Norgren et al. 2003), amyotrophic lateral sclerosis (ALS), and multiple sclerosis (MS) (Malmestrom et al. 2003; Rosengren et al. 1996). In HIV infection, CSF NFL levels are elevated both in early and later infection in individuals with and without neurocognitive impairment (Jessen Krut et al. 2014; Peluso et al. 2013), although levels are highest in HAD (Abdulle et al. 2007; Gisslen et al. 2007; Mellgren et al. 2007). Moreover, CSF NFL levels increase with cART interruption and decrease with cART initiation (Abdulle et al. 2007); neurocognitive performance improves in HAD in parallel with CSF NFL drop (Mellgren et al. 2007). Phosphorylated NFH (pNFH), a more protease-resistant NF, has also been examined in multiple pathologies involving neuronal injury. Specifically, serum pNFH is elevated in amyotrophic lateral sclerosis (ALS) (Ganesalingam et al. 2011), optic neuritis (Pasol et al. 2010), and following acute ischemic stroke (Sellner et al. 2011). In addition, CSF and serum NFH concentrations correlate with each other in ALS (Ganesalingam et al. 2011). Neither CSF nor serum/plasma pNFH levels have been examined in HIV infection. In multiple sclerosis, CSF pNFH but not CSF NFL levels are higher in progressive patients compared with those in relapsing/remitting patients. However, CSF NFL but not CSF pNFH levels predict early clinical disease progression from a first-time demyelinating attack to relapsing/remitting disease (Gresle et al. 2011; Petzold et al. 2005). Therefore, elevations in CSF NFL and pNFH, while both reflecting axonal damage, might have different associations with disease subtypes and/or disease progression, thus supporting separate investigation of each neurofilament subunit in HIV-infected individuals.

Positive correlations between CSF NFL and CSF neopterin (a marker of intrathecal monocyte/macrophage activation) have been described in individuals with HAD (Abdulle et al. 2007; Mellgren et al. 2007), supporting previous evidence that axonal degeneration is associated with such activation (Ryan et al. 2001). Although plasma sCD163 has been correlated with HAND subtype (Burdo et al. 2013), the relationship between plasma monocyte activation markers, CSF NF isoforms, and HAND subtype has not been defined. Among the monocyte activation markers is membrane-bound CD14, a receptor for lipopolysaccharide. Activation of CD14 is associated with CD14 cleavage and release from the cell membrane in a soluble form (sCD14). Notably, plasma sCD14 levels independently predict mortality (Ryan et al. 2001; Sandler et al. 2011) and impaired neurocognitive test performance in HIV-infected subjects (Lyons et al. 2011). In addition, plasma sCD14 is elevated in HIV-infected subjects with cerebral atrophy compared to those without cerebral atrophy (Ryan et al. 2001). Thus, sCD14 is an excellent candidate biomarker of monocyte/macrophage activation associated with HIV-induced neurodegeneration. Similar to CD14, CD163, a monocyte-associated hemoglobin/haptoglobin complex scavenger receptor, is cleaved and shed from activated monocyte/macrophages in a soluble form, sCD163 in inflammatory states (Møller 2012). Plasma sCD163 levels are elevated HIV-infected subjects, particularly those with cognitive impairment (Burdo et al. 2011; Burdo et al. 2013), and plasma levels decrease with cART in parallel with HIV RNA (Burdo et al. 2011). In addition, CD163+ monocyte/macrophages accumulate in perivascular brain regions in individuals with HIV encephalitis (Fischer-Smith et al. 2008a; Roberts et al. 2004). The number of perivascular CD163+ monocyte/macrophages is positively correlated with plasma HIV load (Fischer-Smith et al. 2008b), suggesting trafficking of peripherally activated monocytes to perivascular areas in the brain. Finally, elevated plasma sCD163 levels have been demonstrated in cognitively impaired HIV-infected individuals compared with non-impaired individuals (Burdo et al. 2013), although whether such levels are correlated with expression of markers of neurodegeneration is not known.

To determine the relationship between neurodegeneration and monocyte/macrophage activation in HAND, as assessed by soluble biomarkers, we determined the correlations among levels of CSF neurofilament isoforms (NFL, pNFH), sCD14, and sCD163 across different stages of HAND and cognitively normal HIV-infected adult controls. We then examined associations of soluble biomarker levels with markers of systemic infection (plasma HIV-1 RNA viral load, CD4+ T-lymphocyte count) and with global and domain-specific cognitive impairment and HAND stage.

## Methods

### Study design and setting

We performed a retrospective cross-sectional study using data and biological samples (plasma, CSF) from 48 HIV-infected adults enrolled in the CNS HIV Anti-Retroviral Therapy Effects Research (CHARTER) cohort of the NIMH/NINDS/NIH. Characteristics of the CHARTER cohort are described elsewhere (Heaton et al. 2010). CHARTER is an ongoing, observational cohort study of HIV-infected persons enrolled between 2003 and 2007 from six US university-affiliated HIV treatment centers. Enrolling 1561 HIV+ subjects at baseline, the study was designed to assess the frequency and severity of HAND and the specific contributions of HIV versus HIV-associated comorbidities to neurocognitive impairment. Inclusion criteria for the CHARTER study were broad, but individuals with severe comorbid psychiatric, medical, or neurological disorders deemed likely to adversely affect cognitive functioning were excluded, as were HIV-negative subjects. Our study used data and samples from 48 CHARTER subjects (15 each from ANI and MND subgroups, 3 HAD subjects, and 15 neurocognitively normal (NCN) HIV-infected subjects). The cohort included men and women aged 18–65 years who were ART naïve or currently off of ART and who underwent successful lumbar puncture, venipuncture, and neuropsychological testing. Subjects with a history of CNS opportunistic infection, trauma, epilepsy, MS, known causes of mental retardation, dementia, or active psychotic illness were excluded.

### Data collection

Original data collection for the CHARTER cohort was approved by the Human Subjects Protection Committees of each participating institution. All subjects provided written consent to participate in the CHARTER study. Data were originally obtained through comprehensive neuromedical, neurocognitive, psychiatric, and functional evaluations and collection of blood and urine samples. Collection of CSF was through lumbar puncture (Heaton et al. 2010). The de-identified data and biological samples for this substudy were obtained with permission of the CHARTER steering committee. Because the dataset and samples were de-identified and because our substudy did not involve patient contact, The Children’s Hospital of Philadelphia institutional review board determined (November 21, 2012) that this study did not qualify as human subject research.

### Laboratory assessments

HIV infection was diagnosed by ELISA with Western blot confirmation. Clinical laboratory assessments, including complete blood counts, chemistry panels, rapid plasma regain (RPR), hepatitis C virus (HCV) antibody, and flow cytometry for CD4+ T-lymphocyte count were performed at each CHARTER site’s Clinical Laboratory Improvement Amendments (CLIA)-certified, or CLIA equivalent, medical center laboratory. Plasma HIV loads (viral loads) were quantified by RT-PCR ultrasensitive assay (nominal lower quantitation limit 50 copies/mL; Amplicor®, Roche Diagnostic Systems, Indianapolis, IN) in a central lab (Heaton et al. 2010).

Biomarkers were measured in triplicate by validated, commercially available 96-well plate ELISAs on stored, frozen samples (−80C). Paired CSF/plasma samples were assayed for NFL (UmanDiagnostics AB, limit of detection 31 ng/mL) and pNFH (Biovendor catalog number RD 191138300R, limit of detection 23.5 pg/mL); NFL and pNFH were detected only in CSF samples. sCD14 and sCD163 were assayed in paired CSF/plasma samples (R&D Systems, catalog number DC140, limit of detection 125 pg/mL sCD14 and Trillium Diagnostics, IQP-383, limit of detection 0.23 ng/mL sCD163).

### Neurocognitive assessments

All CHARTER study subjects completed a comprehensive neuropsychological test battery assessing seven cognitive domains commonly affected in HIV infection (verbal fluency, executive functioning, speed of information processing, learning, recall, working memory, and motor skills). Raw test scores were converted to demographically adjusted T-scores using the best available normative data accounting for age, sex, ethnicity, and education. Functional impairment was assessed using the Patient’s Assessment of Own Functioning Inventory (PAOFI) and instrumental activities of daily living (IADL) questionnaire (Heaton et al. 2010). A global performance score was determined as previously described (Carey et al. 2004; Woods et al. 2004). HAND status was classified according to Frascati criteria (Antinori et al. 2007).

### Data analysis and statistical methods

Data were analyzed using Stata version 12.1 (StataCorp, College Station, Texas, 2011). Continuous variables were described using median and intraquartile range (IQR), and intergroup differences were evaluated using Kruskal-Wallis tests and Wilcoxan rank-sum tests. Categorical variables were described using counts and percents, and intergroup differences were compared using the chi-square test. Spearman’s correlation coefficients were used for correlations between biomarkers. Statistical significance was determined a prior as a two-tailed *p* value <0.05.

## Results

To avoid any potential confounding effects of ART on expression of selected biomarkers of inflammation and neuronal injury, we selected a study cohort of individuals currently not receiving ART. Cohort demographics, medical, and laboratory characteristics are summarized in Table 1. Among the 48 subjects examined, 75 % were male, and 53 % were African American. The median age was 39.5 (IQR 36–47.5, range 19–62). Only plasma CD4+ T-lymphocyte count and plasma HIV-1 RNA differed across HAND subtypes: CD4+ T-lymphocyte count was lower and plasma HIV-1 RNA was higher in HAD when compared with ANI, MND, and neurocognitively normal controls (*p* = 0.046, *p* = 0.038, respectively). Thus, as expected, our subjects with HAD demonstrated greater immune deficiency and poorer systemic viral control than those without HAD as has been previously demonstrated in ART-naïve patients (McArthur and Brew 2010; McArthur et al. 2010). Finally, only one subject (ANI) was actively using illicit drugs (marijuana, opioids) during this study.

**Table 1:** Demographic and clinical characteristics of the study population

Characteristic^a^	All, *n* = 48	NCN, *n* = 15	ANI, *n* = 15	MND, *n* = 15	HAD, *n* = 3	*p* ^b^
Male sex	36 (75)	12 (80)	9 (60)	12 (80)	3 (100)	0.362
Black race	25 (53)	8 (57)	7 (47)	9 (60)	1 (33)	0.777
Age (years)	39.5 (36–47.5)	44 (36–49)	38 (31–40)	40 (35–48)	47 (38–50)	0.392
Duration of HIV (months)	98 (29–157)	131 (71–186)	99 (11–122)	98 (23–162)	34 (2–234)	0.616
Education (years)	12 (11–13)	13 (12–15)	11 (10–12)	12 (9–14)	13 (12–14)	0.102
HCV positive	14 (31)	5 (33)	6 (40)	3 (20)	0 (0)	0.420
RPR positive	5 (10)	1 (7)	1 (7)	3 (21)	0 (0)	0.538
CD4 nadir ≤200	14 (29)	5 (33)	3 (20)	4 (27)	2 (67)	0.420
Plasma CD4 <50	5 (10)	1 (7)	1 (7)	1 (7)	2 (67)	0.046
50–199	5 (10)	1 (7)	2 (13)	2 (13)	0 (0)	–
200–349	7 (15)	0 (0)	3 (20)	3 (20)	1 (33)	–
≥350	31 (65)	13 (87)	9 (60)	9 (60)	0 (0)	–
Plasma HIV–1 RNA (log_10_ copies/mL)	4.34 (3.66–4.87)	3.83 (3.15–4.06)	4.68 (3.97–5.04)	4.54 (3.93–5.08)	5.53 (3.12–6.16)	0.038
CSF HIV–1 RNA (log_10_ copies/mL)	2.45 (1.70–3.20)	1.83 (1.70–3.20)	2.84 (1.70–3.20)	2.86 (2.01–3.41)	1.70 (1.70–2.35)	0.330

^a^Categorical variables are described using *n* (%). Continuous variables are described using median (IQR)

^b^ *p* values to compare characteristics among different subgroups of HAND were calculated using chi-square tests for categorical variables and Kruskal-Wallis tests for continuous variables

**Fig. 1:**
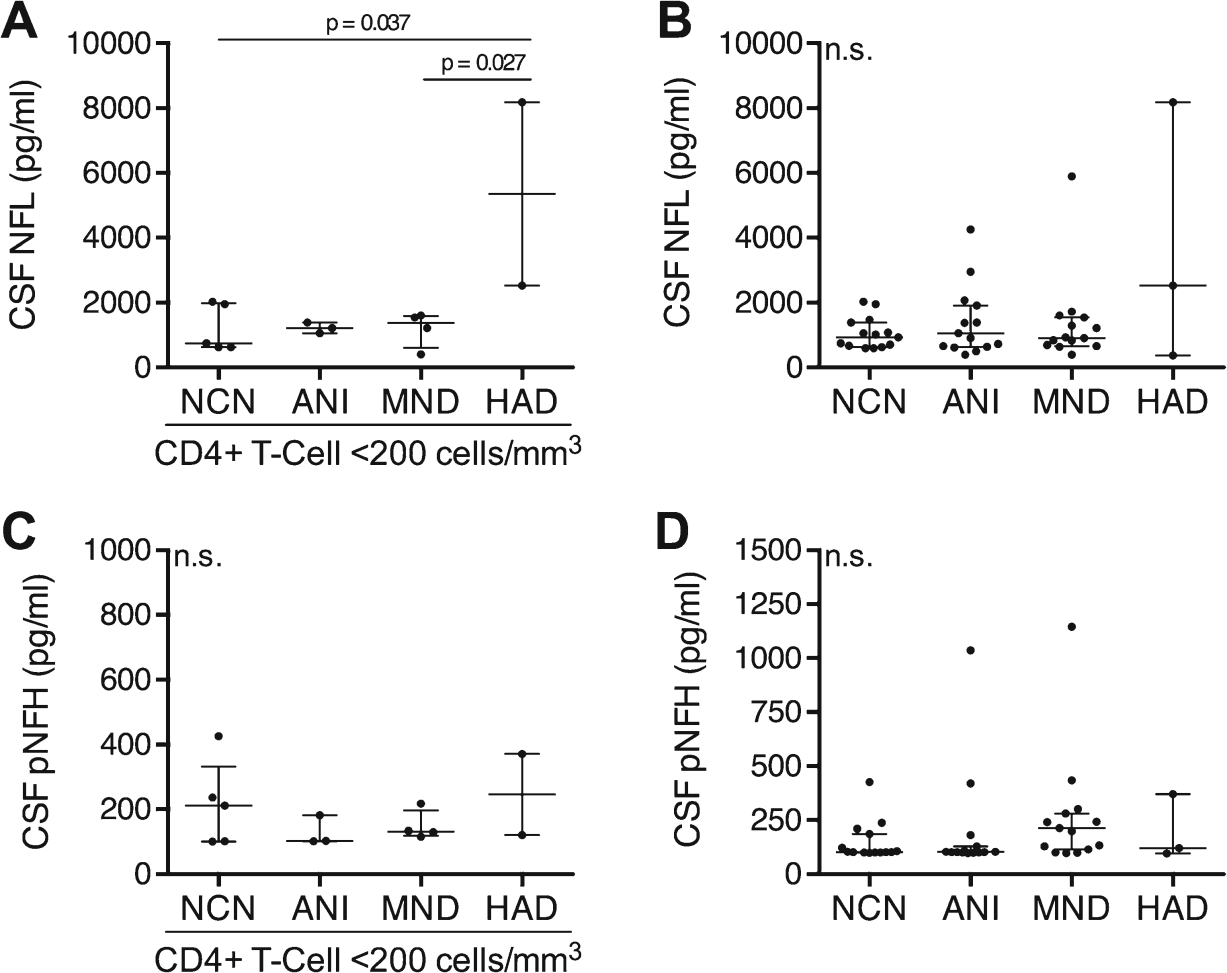
CSF NFL is elevated in individuals with HAD and a history of immunosuppression. NFL concentrations in the CSF of HIV+ **a** individuals with CD4 count nadir ≤200 and **b** individuals with any CD4 count nadir. pNFH concentrations in the CSF of HIV+ **c** individuals with CD4 count nadir ≤200 and **d** individuals with any CD4 count nadir. Data is presented as median and IQR with differences between groups evaluated using Kruskal-Wallis tests

To determine the associations between neuronal injury, monocyte/macrophage activation, and the severity of HAND, we first examined expression of neurofilament isoforms NFL and pNFH in CSF and expression of sCD163 and sCD14 in both CSF and plasma across the different sub-types of HAND. Among individuals with CD4+ T-lymphocyte nadirs ≤200, CSF NFL levels were significantly elevated in individuals with HAD compared with neurocognitively normal subjects and those with MND (Fig. 1a). There were no differences among HAND subtypes in levels of CSF NFL across all CD4+ T-lymphocyte nadirs (Fig. 1b), CSF pNFH (Fig. 1c, d), CSF/plasma sCD14, or CSF/plasma CD163 (data not shown). In addition, CSF NFL levels did not significantly differ across HAND subtypes by current CD4+ T-lymphocyte count among neurocognitively normal subjects in contrast to another recent study (Peterson et al. 2013). Finally, because our cohort did not include HIV-negative individuals, we compared absolute NFL levels with historical age-specific controls using the same NFL ELISA assay (UmanDiagnostics, AB) (Jessen Krut et al. 2014). Using these historical controls, we found that 10/15 neurocognitively normal, 11/15 ANI, 11/15 MND, and 2/3 HAD subjects demonstrated elevated CSF NFL levels, suggesting ongoing subclinical neuronal injury in HIV-infected individuals regardless of neurocognitive status.

**Fig. 2:**
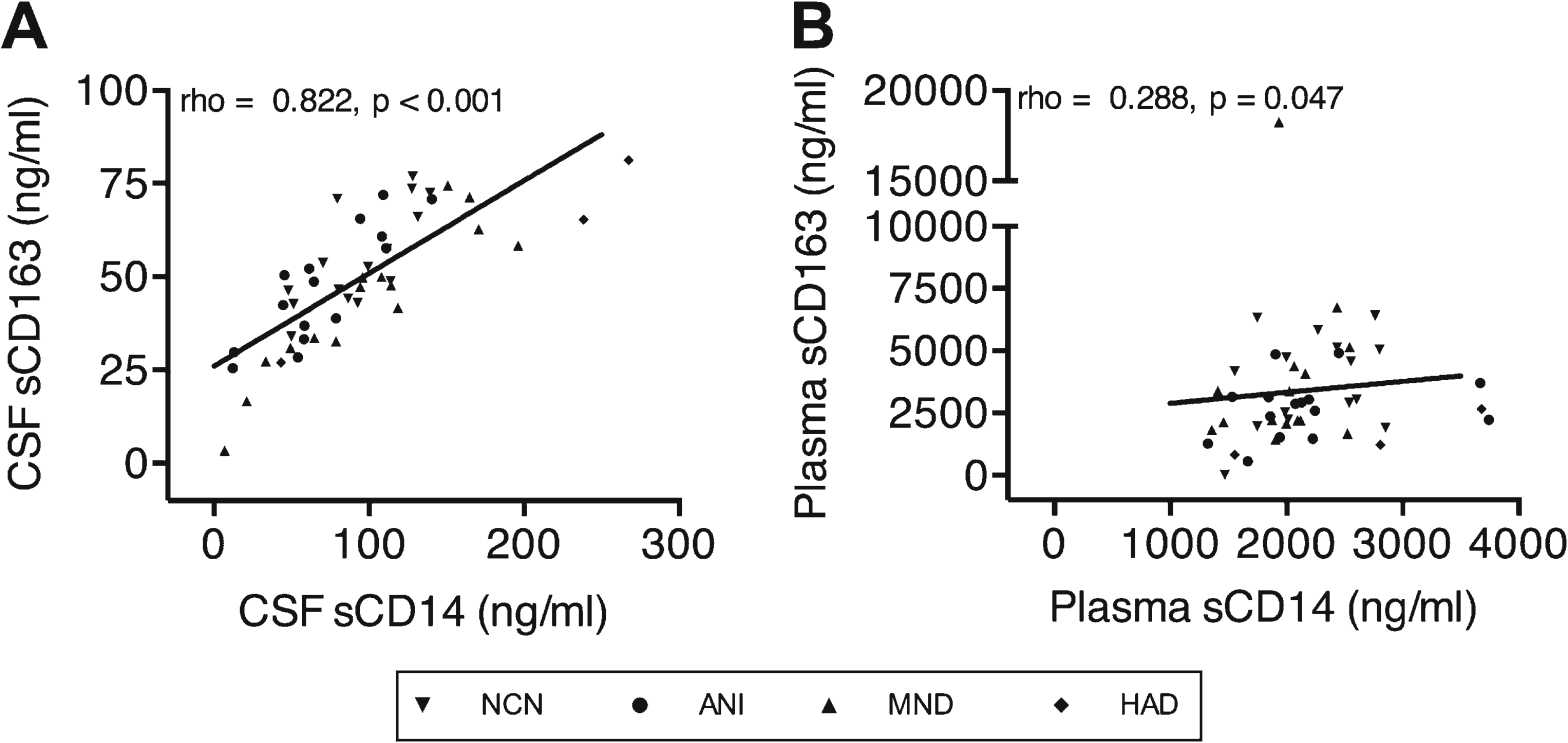
Monocyte activation markers intra-compartmentally correlate within the CSF and plasma. Correlations between the monocyte markers sCD163 and sCD14 within the **a** CSF and **b** plasma across all HIV-infected individuals with and without HAND. Correlations were analyzed using Spearman’s correlation coefficients

Both CSF and plasma expression of sCD163 and sCD14 correlated within compartments (Fig. 2a, b), as did CSF NFL and pNFH (data not shown). The only other similar intra-compartment correlations of monocyte activation markers we are aware of in the HIV literature have demonstrated a correlation between plasma sCD163 and CSF neopterin (Burdo et al. 2013) and plasma sCD163 and plasma sCD14 (Burdo et al. 2011). The strength and significance of the correlation shown in Fig. 2 suggests that both sCD163 and sCD14 similarly reflect monocyte activation in the CSF (and either may be used with confidence as a CSF marker of monocyte activation) and supports the conclusion that these markers are predictably detecting monocyte/macrophage activation and neuronal injury in these subjects.

**Fig. 3:**
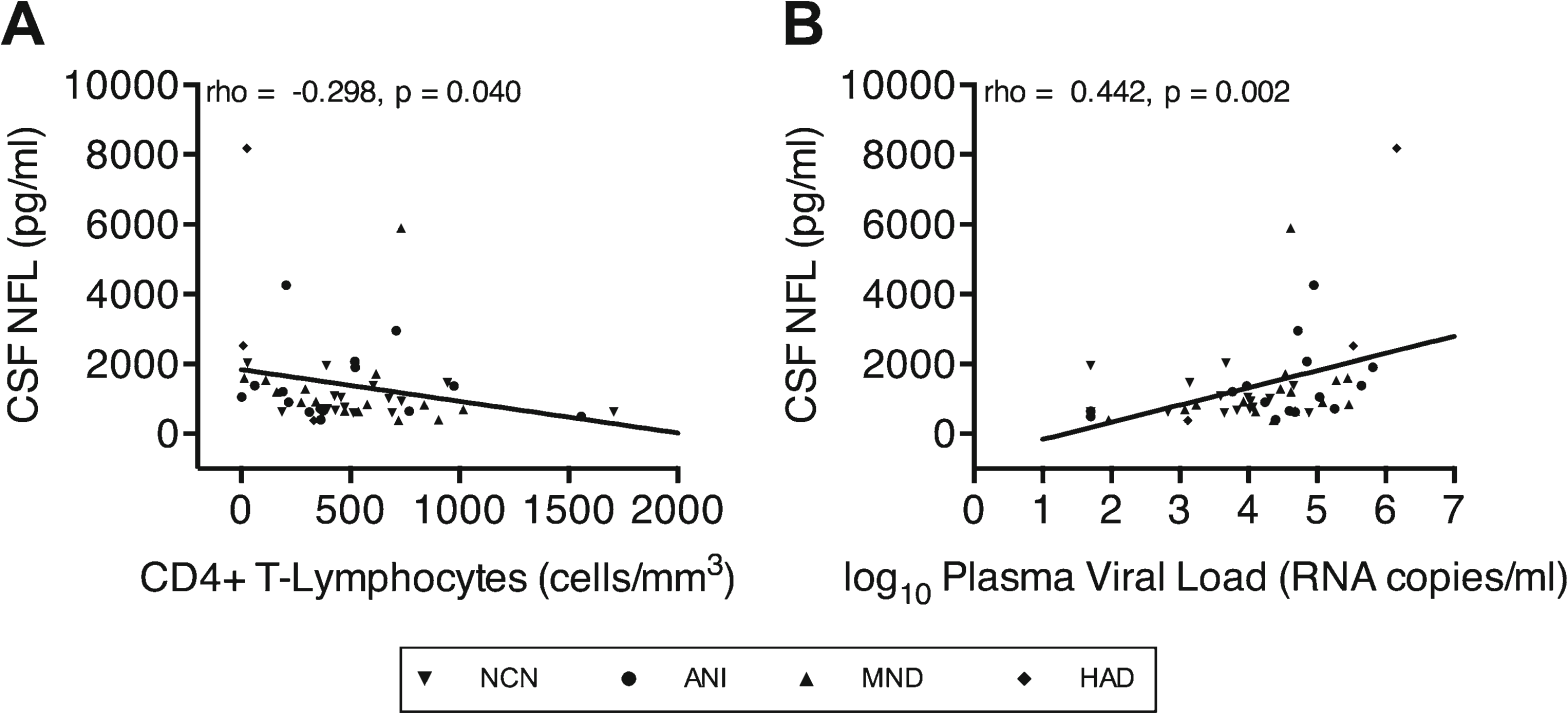
CSF NFL correlates negatively with plasma CD4+ T-lymphocyte count and positively with HIV-1 RNA load. Correlations between CSF NFL concentrations and plasma **a** CD4+ T-lymphocyte count and **b** HIV-1 RNA load in all HIV-infected individuals with and without HAND. Correlations were analyzed using Spearman’s correlation coefficients

**Fig. 4:**
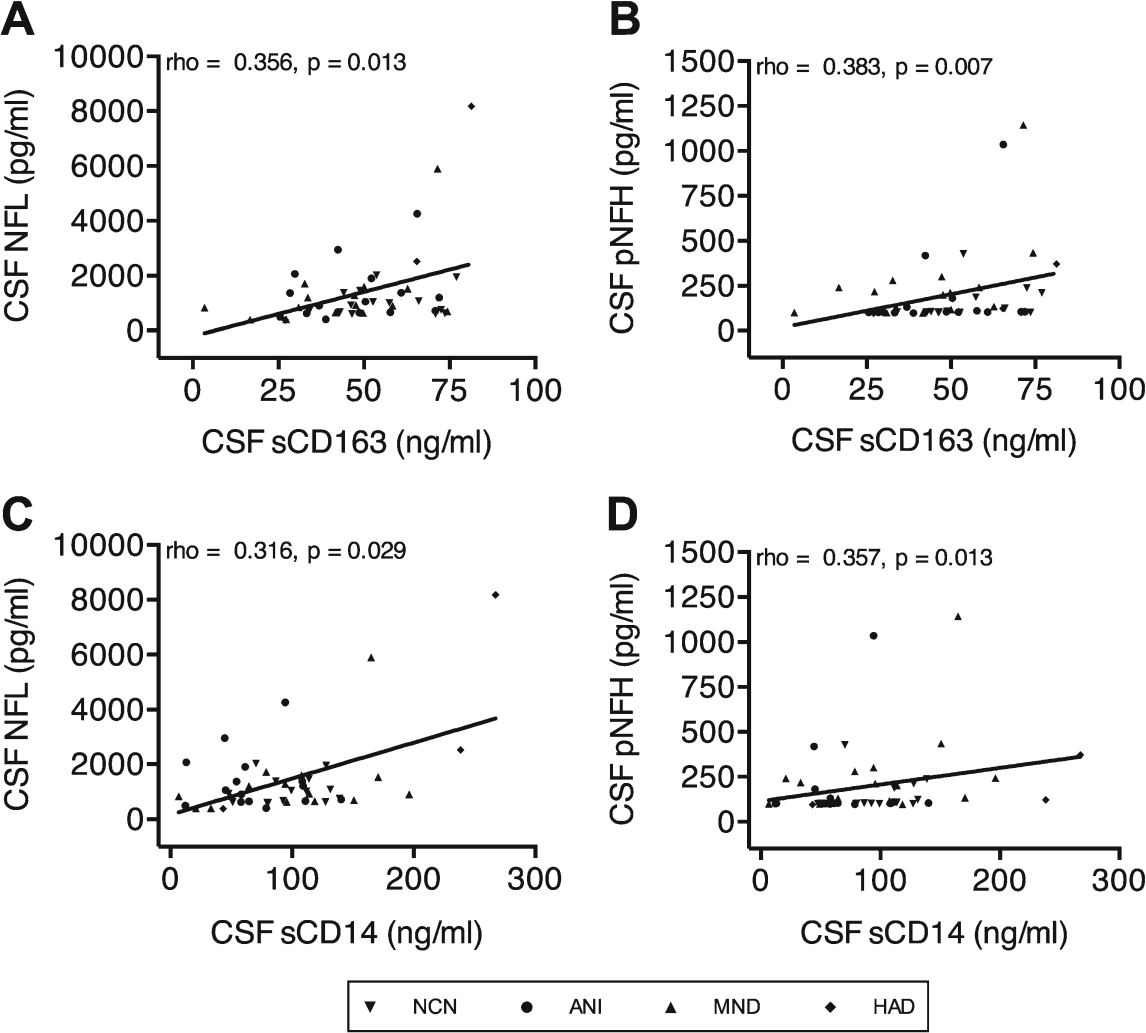
Neurofilament concentrations positively correlate with monocyte activation markers within the CSF. Correlations between sCD163 and **a** NFL and **b** pNFH and between sCD14 and **c** NFL and **d** pNFH within the CSF of all HIV-infected individuals with and without HAND. Correlations were analyzed using Spearman’s correlation coefficients

Next, to assess the relationship between CSF markers of neurodegeneration, monocyte activation, and systemic HIV infection and to determine whether markers of monocyte/macrophage activation correlate with neuronal injury in HAND, each of the above candidate biomarkers was examined in association with each other and with plasma HIV-1 RNA viral load and CD4+ T-lymphocyte count in all subjects, regardless of cognitive impairment. Here, CSF NFL was negatively correlated with CD4+ T-lymphocyte count (Fig. 3a) and positively correlated with plasma HIV-1 RNA viral load (Fig. 3b), suggesting a relationship between neuronal damage and systemic HIV infection. CSF pNFH did not demonstrate the same correlations with markers of systemic HIV infection. Additionally, CSF NFL and pNFH levels correlated strongly and positively with CSF sCD163 (Fig. 4a, b) and CSF sCD14 (Fig. 4c, d), thus indicating that monocyte/macrophage activation within the CNS compartment is tightly linked to neuronal injury. In contrast to CSF expression, plasma expression of sCD14 and sCD163 did not correlate with CSF NFL or CSF pNFH. This suggests either no association between systemic monocyte/macrophage activation and CNS neuronal injury in this untreated cohort or a low level of sensitivity of plasma sCD14 and sCD163 detection as biomarkers for CNS neuronal injury in comparison with CSF sCD14 and sCD163.

Examination of biomarker associations with cognitive testing subdomain and global cognitive scores demonstrated that CSF pNFH was significantly higher in subjects with impaired speed of information processing (median CSF pNFH 103 pg/mL in unimpaired compared to 371 pg/mL in impaired subjects, total CSF pNFH range 96–1144 pg/mL, *p* = 0.018) and memory (median CSF pNFH 103 pg/mL in unimpaired compared to 220 pg/mL in impaired subjects, *p* = 0.021) compared to unimpaired subjects, although the magnitude of this effect was small. There were no significant correlations among NFL, plasma/CSF sCD14, or sCD163 and subdomain global deficit scores or impairment (data not shown).

Finally, to investigate whether hepatitis C virus (HCV) serostatus affected the relationships described above between the neurofilament isoforms, monocyte activation markers, and HAND subtypes, we performed an exploratory analysis examining concentrations of each of these markers across HAND for HCV+ and HCV− subjects separately. No statistically significant differences were found (data not shown). To determine whether a differential level of peripheral or central monocyte activation was present in HCV+ versus HCV− subjects, we then compared monocyte activation marker concentrations between these populations. After manually removing outlier values, none of the markers examined was significantly different between HCV+ and HCV− subjects (data not shown).

## Discussion

We have demonstrated a strong correlation between CSF markers of neuronal damage (NFL, pNFH) and monocyte/macrophage activation (sCD14 and sCD163) in an unselected cohort of viremic HIV-infected individuals not receiving ART. No correlations between CSF NFL/pNFH and plasma sCD14 and sCD163 were demonstrated. In addition, plasma sCD163 did not vary significantly across different HAND subtypes, in contrast with a previous study of virologically suppressed individuals on ART (Burdo et al. 2013). However, plasma sCD163 levels in our cohort were higher than those observed in the previous study, which could reflect other factors contributing to monocyte/macrophage activation in this cohort, including perhaps uncontrolled peripheral HIV infection, coinfection with HCV, or syphilis in some individuals (Table 1). Notably, there were no observed differences which correlated with HCV serostatus in this cohort. Nonetheless, our study clearly demonstrates that CNS monocyte/macrophage activation, as measured by CSF sCD163 and sCD14 is strongly correlated with neuronal injury, and based on current understanding of HIV neuropathophysiology, this likely represents a causal association.

The possible association between CSF neurofilaments and severity of cognitive impairment in HIV-infected individuals has not been fully described and involves complex and numerous variables. Similar to others (Abdulle et al. 2007; Gisslen et al. 2007; Mellgren et al. 2007), we found that CSF NFL was significantly elevated in HAD subjects compared with those with milder (MND) or no cognitive dysfunction; this relationship was observed only in individuals with CD4 count nadirs ≤200, which is strongly associated with an increased risk for HAND (Heaton et al. 2010; McArthur and Brew 2010; McArthur et al. 2010). Recent studies have demonstrated significant elevations in CSF NFL in individuals with acute HIV infection and in HIV-infected individuals without cognitive impairment (Jessen Krut et al. 2014; Peluso et al. 2013), although higher levels are observed in individuals with HAD, the most severe form of HAND. Elevated CSF NFL in acute HIV infection has also been associated with low N-acetylaspartate/creatine ratios (another indicator of neuronal injury) in cortex and white matter, as measured by brain magnetic resonance spectroscopy (Peluso et al. 2013). In contrast, our study failed to demonstrate significant elevations of CSF NFL in ANI and MND individuals when compared with neurocognitively normal HIV-infected individuals, although we do not have a seronegative control group to determine whether CSF NFL is elevated in our seropositive neurocognitively normal control group. In examining previously published age-specific normative data from 107 HIV-uninfected subjects using the same ELISA platform (Jessen Krut et al. 2014), it does indeed appear that a significant proportion of our neurocognitively normal, ANI, and MND subjects had NFL elevations compared to uninfected controls. These data support the conclusion that some untreated subjects have ongoing subclinical CNS injury. This conclusion is inherently limited by the use of historical controls. Thus, although CSF NFL can be considered to be sensitive marker of neuronal injury in the CNS in HIV infection, whether it reliably predicts or correlates with the severity of neuronal injury and neurocognitive impairment remains undefined. This is clearly an important gap in our understanding of the significance of the relative degree of neuronal injury in determining the presence and/or severity of symptoms of cognitive dysfunction in HIV infection.

Our data suggest that CSF pNFH levels in individuals with HAND differ in comparison with CSF NFL levels. While CSF pNFH did not vary significantly across the different HAND sub-types, on cognitive testing subdomain assessment, we demonstrated significant associations with CSF pNFH and impaired speed of information processing and memory. However, the magnitude of effect was small. Speed of information processing is dependent on multiple neurologic pathways and therefore may be nonspecifically affected compared to other, more localizable subdomains. In addition, CSF pNFH had an even stronger correlation with CSF markers of monocyte activation compared to CSF NFL, suggesting that these different neurofilament isoforms may represent distinct neuronal injury pathways with ultimately different clinical implications.

Our study showed no significant correlation between CSF NFL level and current CD4 count among neurocognitively normal HIV-infected subjects as demonstrated in previous studies (Abdulle et al. 2007; Peterson et al. 2013), perhaps reflecting a sample size too small to stratify into CD4 subgroup ranges. However, our data was significant for a negative correlation between CSF NFL and plasma CD4+ T-lymphocyte count across all strata of cognitive impairment, as well as a positive correlation between CSF NFL and plasma HIV-1 RNA viral load. Taken together, these data suggest more neuronal injury in more advanced (or poorly controlled) systemic infection and may suggest a part of a mechanistic link to the clinical observation that low CD4+ T-lymphocyte count and high viral load are risk factors for HAD in untreated HIV infection. Notably, there was not a similar correlation between CSF pNFH and the same plasma markers of systemic HIV infection.

Because identifying a valid plasma biomarker for neuronal injury and/or HAND in HIV-infected individuals is still an unmet need, we considered the potential usefulness of neurofilament isoforms as such biomarkers. Published studies indicate that NFL is quickly proteolytically degraded in blood specimens, while pNFH is more protease resistant (Gresle et al. 2011). Based on prior investigations demonstrating serum pNFH elevation in other neurodegenerative diseases (Ganesalingam et al. 2011; Pasol et al. 2010; Sellner et al. 2011), serum pNFH could serve as a biomarker for neurodegeneration in the brain and/or spinal cord, each of which is affected in many individuals with HIV infection. Analyses of serum neurofilament isoforms in HIV infection have not been reported. However, despite demonstrating pNFH in CSF samples from our cohort, we failed to detect pNFH in matched plasma samples.

About one third of our overall study population was HCV positive, consistent with other estimates in adult HIV-infected populations (Letendre et al. 2005; Sherman et al. 2014). However, HCV was not present in any subject with HAD. Prior literature suggests that individuals co-infected with both HIV and HCV have worsened cognitive impairment compared to HIV infection alone (Clifford et al. 2005; Garvey et al. 2012). Mechanistically, HCV replicates in CD68+ macrophages of autopsied HIV-infected brain tissue (Wilkinson et al. 2009) and is associated with increased macrophage expression of pro-inflammatory cytokines (Wilkinson et al. 2010). Coinfection with HCV and HIV may therefore affect neurocognitive status via its effects on monocyte/macrophage activation (Gill and Kolson 2014). However, in this study, we did not observe a difference in monocyte activation marker concentrations between HCV+ and HCV− subjects, nor did we observe a difference in neurofilament isoform concentrations or monocyte activation marker concentrations in HCV+ and HCV− subjects when analyzed across the spectrum of HAND. Whether these findings are truly representative of HIV/HCV coinfection neuropathology, or if they were limited by our sample size or another confounding factor not accounted for in these associations is unclear.

Our study had several limitations. First, because ART is now widely distributed, recruiting ART-free individuals is particularly difficult; our study therefore included a small cohort size (*n* = 48) with few HAD subjects (*n* = 3). Thus, sub-analyses with even smaller numbers (e.g., the relationship between HAND and CSF NFL among patients with CD4 count nadir <200) may be underpowered and have limited potential significance. These data are consistent with other prior reports though, and the possible effect of a profound immunosuppression on these relationships requires further investigation. Second, because this was a cross-sectional correlation study, factors confounding the relationship between monocyte/macrophage activation and HAND status are not accounted for in statistical associations. However, relevant covariates were equivalent across the different subtypes of HAND, except for CD4+ T-lymphocyte count and viral load, which are known risk factors for HAD. In addition, causality cannot be inferred in a cross-sectional study. Finally, by including only HIV-infected viremic subjects not receiving ART, we might obscure relevant associations between plasma monocyte/macrophage activation and neurodegeneration because of high levels of plasma monocyte/macrophage activation in untreated, viremic subjects. Nonetheless, we have eliminated the possibility of unanticipated effects of ART on inflammation and neurodegeneration.

In summary, we have demonstrated a positive correlation between expression of CSF neurofilament isoforms and CSF sCD14 and sCD163 in viremic HIV-infected individuals not receiving ART, thus directly linking CNS monocyte/macrophage activation with neuronal injury. Future studies are necessary to determine whether this relationship is specific to HIV neuropathogenesis, or if it is observed in other neuroinflammatory disorders as well. Furthermore, sCD14 and sCD163 levels are highly correlated in CSF and plasma, suggested that monocyte/macrophage activation can reliably be detected by ELISA in such stored tissue specimens. We have also confirmed that severe CD4 depletion (a risk factor for HAD) is associated with elevated CSF NFL levels at the time of diagnosis of HAD and that elevated CSF NFL levels are highly correlated with low CD4+ T-lymphocyte counts and high plasma HIV-1 RNA viral load, suggesting a link between neuronal injury and systemic HIV infection. A previous study has suggested that CSF NFL can serve as a *predictive* biomarker for HAD in individuals not receiving ART (Gisslen et al. 2007). Whether CSF NFL is a sensitive predictor of risk for HAND subtypes, and whether sCD14 and sCD163 might also have predictive value on later development/progression of HAND in viremic versus aviremic ART-experienced individuals remains to be determined. Finally, we have demonstrated somewhat different correlations of NFL and pNFH with respect to cognitive measurements, suggesting that the different neurofilament isoforms may represent distinct neuronal injury pathways in HIV neuropathophysiology.

## References

[CR1] Abdulle S, Mellgren A, Brew BJ, Cinque P, Hagberg L, Price RW, Rosengren L, Gisslén M (2007). CSF neurofilament protein (NFL)—a marker of active HIV-related neurodegeneration. J Neurol.

[CR2] Antinori A, Arendt G, Becker JT, Brew BJ, Byrd DA, Cherner M, Clifford DB, Cinque P, Epstein LG, Goodkin K, Gisslen M, Grant I, Heaton RK, Joseph J, Marder K, Marra CM, McArthur JC, Nunn M, Price RW, Pulliam L, Robertson KR, Sacktor N, Valcour V, Wojna VE (2007). Updated research nosology for HIV-associated neurocognitive disorders. Neurology.

[CR3] Burdo TH, Lentz MR, Autissier P, Krishnan A, Halpern E, Letendre S, Rosenberg ES, Ellis RJ, Williams KC (2011). Soluble CD163 made by monocyte/macrophages is a novel marker of HIV activity in early and chronic infection prior to and after anti-retroviral therapy. J Infect Dis.

[CR4] Burdo TH, Weiffenbach A, Woods SP, Letendre S, Ellis RJ, Williams KC (2013). Elevated sCD163 is a marker of neurocognitive impairment in HIV infection. AIDS.

[CR5] Carey CL, Woods SP, Gonzalez R, Conover E, Marcotte TD, Grant I, Heaton RK (2004). Predictive validity of global deficit scores in detecting neuropsychological impairment in HIV infection. J Clin Exp Neuropsychol.

[CR6] Cherner M, Masliah E, Ellis RJ, Marcotte TD, Moore DJ, Grant I, Heaton RK (2002). Neurocognitive dysfunction predicts postmortem findings of HIV encephalitis. Neurology.

[CR7] Clifford DB, Evans SR, Yang Y, Gulick RM (2005). The neuropsychological and neurological impact of hepatitis C virus co-infection in HIV-infected subjects. AIDS.

[CR8] Ellis R, Langford D, Masliah E (2007). HIV and antiretroviral therapy in the brain: neuronal injury and repair. Nat Rev Neurosci.

[CR9] Ellis RJ, Deutsch R, Heaton RK, Marcotte TD, McCutchan JA, Nelson JA, Abramson I, Thal LJ, Atkinson JH, Wallace MR, Grant I (1997). Neurocognitive impairment is an independent risk factor for death in HIV infection. San Diego HIV neurobehavioral research center group. Arch Neurol.

[CR10] Fischer-Smith T, Bell C, Croul S, Lewis M, Rappaport J (2008). Monocyte/macrophage trafficking in acquired immunodeficiency syndrome encephalitis: lessons from human and nonhuman primate studies. J Neurovirol.

[CR11] Fischer-Smith T, Tedaldi EM, Rappaport J (2008). CD163/CD16 coexpression by circulating monocytes/macrophages in HIV: potential biomarkers for HIV infection and AIDS progression. AIDS Res Hum Retrovir.

[CR12] Ganesalingam J, An J, Shaw CE, Shaw G, Lacomis D, Bowser R (2011). Combination of neurofilament heavy chain and complement C3 as CSF biomarkers for ALS. J Neurochem.

[CR13] Garvey LJ, Pavese N, Ramlackhansingh A, Thomson E, Allsop JM, Politis M, Kulasegaram R, Main J, Brooks DJ, Taylor-Robinson SD, Winston A (2012). Acute HCV/HIV coinfection is associated with cognitive dysfunction and cerebral metabolite disturbance, but not increased microglial cell activation. PLoS One.

[CR14] Gelman BB, Chen T, Lisinicchia JG, Soukup VM, Carmical JR, Starkey JM, Masliah E, Commins DL, Brandt D, Grant I, Singer EJ, Levine AJ, Miller J, Winkler JM, Fox HS, Luxon BA, Morgello S (2012). The National NeuroAIDS Tissue Consortium brain gene array: two types of HIV-associated neurocognitive impairment. PLoS One.

[CR15] Gill AJ, Kolson DL (2014). Chronic inflammation and the role for cofactors (hepatitis C, drug abuse, antiretroviral drug toxicity, aging) in HAND persistence. Curr HIV/AIDS Rep.

[CR16] Gisslen M, Hagberg L, Brew BJ, Cinque P, Price RW, Rosengren L (2007). Elevated cerebrospinal fluid neurofilament light protein concentrations predict the development of AIDS dementia complex. J Infect Dis.

[CR17] Grant I, Franklin DR, Deutsch R, Woods SP, Vaida F, Ellis RJ, Letendre SL, Marcotte TD, Atkinson JH, Collier AC, Marra CM, Clifford DB, Gelman BB, McArthur JC, Morgello S, Simpson DM, McCutchan JA, Abramson I, Gamst A, Fennema-Notestine C, Smith DM, Heaton RK (2014). Asymptomatic HIV-associated neurocognitive impairment increases risk for symptomatic decline. Neurology.

[CR18] Gresle MM, Butzkueven H, Shaw G (2011). Neurofilament proteins as body fluid biomarkers of neurodegeneration in multiple sclerosis. Mult Scler Int.

[CR19] Heaton R, Franklin D, Woods S, Marra C, Clifford D, Gelman B, McArthur J, Morgello S, McCutchan A, Grant I (2012) Asymptomatic HIV-associated Neurocognitive Disorder (ANI) Increases Risk for Future Symptomatic Decline: A CHARTER Longitudinal Study. Abstract #77. In: 19th Conference on Retroviruses and Opportunistic Infections (CROI): Seattle, WA

[CR20] Heaton RK, Clifford DB, Franklin DR, Woods SP, Ake C, Vaida F, Ellis RJ, Letendre SL, Marcotte TD, Atkinson JH, Rivera-Mindt M, Vigil OR, Taylor MJ, Collier AC, Marra CM, Gelman BB, McArthur JC, Morgello S, Simpson DM, McCutchan JA, Abramson I, Gamst A, Fennema-Notestine C, Jernigan TL, Wong J, Grant I, Group C (2010). HIV-associated neurocognitive disorders persist in the era of potent antiretroviral therapy: CHARTER Study. Neurology.

[CR21] Jessen Krut J, Mellberg T, Price RW, Hagberg L, Fuchs D, Rosengren L, Nilsson S, Zetterberg H, Gisslen M (2014). Biomarker evidence of axonal injury in neuroasymptomatic HIV-1 patients. PLoS One.

[CR22] Julien JP (1999). Neurofilament functions in health and disease. Curr Opin Neurobiol.

[CR23] Letendre SL, Cherner M, Ellis RJ, Marquie-Beck J, Gragg B, Marcotte T, Heaton RK, McCutchan JA, Grant I (2005). The effects of hepatitis C, HIV, and methamphetamine dependence on neuropsychological performance: biological correlates of disease. AIDS.

[CR24] Lyons JL, Uno H, Ancuta P, Kamat A, Moore DJ, Singer EJ, Morgello S, Gabuzda D (2011). Plasma sCD14 is a biomarker associated with impaired neurocognitive test performance in attention and learning domains in HIV infection. J Acquir Immune Defic Syndr.

[CR25] Malmestrom C, Haghighi S, Rosengren L, Andersen O, Lycke J (2003). Neurofilament light protein and glial fibrillary acidic protein as biological markers in MS. Neurology.

[CR26] Masliah E, Heaton RK, Marcotte TD, Ellis RJ, Wiley CA, Mallory M, Achim CL, McCutchan JA, Nelson JA, Atkinson JH, Grant I (1997). Dendritic injury is a pathological substrate for human immunodeficiency virus-related cognitive disorders. HNRC group. The HIV neurobehavioral research center. Ann Neurol.

[CR27] McArthur JC, Brew BJ (2010). HIV-associated neurocognitive disorders: is there a hidden epidemic?. AIDS.

[CR28] McArthur JC, Steiner J, Sacktor N, Nath A (2010). Human immunodeficiency virus-associated neurocognitive disorders: mind the gap. Ann Neurol.

[CR29] Mellgren A, Price RW, Hagberg L, Rosengren L, Brew BJ, Gisslén M (2007). Antiretroviral treatment reduces increased CSF neurofilament protein (NFL) in HIV-1 infection. Neurology.

[CR30] Møller HJ (2012). Soluble CD163. Scand J Clin Lab Invest.

[CR31] Norgren N, Rosengren L, Stigbrand T (2003). Elevated neurofilament levels in neurological diseases. Brain Res.

[CR32] Pasol J, Feuer W, Yang C, Shaw G, Kardon R, Guy J (2010). Phosphorylated neurofilament heavy chain correlations to visual function, optical coherence tomography, and treatment. Mult Scler Int.

[CR33] Peluso MJ, Meyerhoff DJ, Price RW, Peterson J, Lee E, Young AC, Walter R, Fuchs D, Brew BJ, Cinque P, Robertson K, Hagberg L, Zetterberg H, Gisslen M, Spudich S (2013). Cerebrospinal fluid and neuroimaging biomarker abnormalities suggest early neurological injury in a subset of individuals during primary HIV infection. J Infect Dis.

[CR34] Peterson J, Zetterberg H, Hagberg L, Spudich S, Gisslen M, Price R (2013) Changing Cerebrospinal Fluid Concentrations of Neurofilament Light Chain Protein, Tau, and Amyloid Proteins Characterize Evolving Central Nervous System Injury in HIV-1 Infection. Abstract #441. In: 20th Conference on Retroviruses and Opportunistic Infections (CROI), Atlanta, GA

[CR35] Petzold A, Eikelenboom MJ, Keir G, Grant D, Lazeron RHC, Polman CH, Uitdehaag BMJ, Thompson EJ, Giovannoni G (2005). Axonal damage accumulates in the progressive phase of multiple sclerosis: three year follow up study. J Neurol Neurosurg Psychiatry.

[CR36] Roberts ES, Masliah E, Fox HS (2004). CD163 identifies a unique population of ramified microglia in HIV encephalitis (HIVE). J Neuropathol Exp Neurol.

[CR37] Rosengren LE, Karlsson JE, Karlsson JO, Persson LI, Wikkelsø C (1996). Patients with amyotrophic lateral sclerosis and other neurodegenerative diseases have increased levels of neurofilament protein in CSF. J Neurochem.

[CR38] Ryan LA, Zheng J, Brester M, Bohac D, Hahn F, Anderson J, Ratanasuwan W, Gendelman HE, Swindells S (2001). Plasma levels of soluble CD14 and tumor necrosis factor-alpha type II receptor correlate with cognitive dysfunction during human immunodeficiency virus type 1 infection. J Infect Dis.

[CR39] Sandler NG, Wand H, Roque A, Law M, Nason MC, Nixon DE, Pedersen C, Ruxrungtham K, Lewin SR, Emery S, Neaton JD, Brenchley JM, Deeks SG, Sereti I, Douek DC, Group ISS (2011). Plasma levels of soluble CD14 independently predict mortality in HIV infection. J Infect Dis.

[CR40] Sellner J, Patel A, Dassan P, Brown MM, Petzold A (2011). Hyperacute detection of neurofilament heavy chain in serum following stroke: a transient sign. Neurochem Res.

[CR41] Sherman KE, Thomas D, Chung RT (2014). Human immunodeficiency virus and liver disease forum 2012. Hepatology.

[CR42] Szaro BG, Strong MJ (2010). Post-transcriptional control of neurofilaments: new roles in development, regeneration and neurodegenerative disease. Trends Neurosci.

[CR43] Tan IL, McArthur JC (2012). HIV-associated neurological disorders: a guide to pharmacotherapy. CNS Drugs.

[CR44] Valcour V, Paul R, Chiao S, Wendelken LA, Miller B (2011). Screening for cognitive impairment in human immunodeficiency virus. Clin Infect Dis.

[CR45] Vivithanaporn P, Heo G, Gamble J, Krentz HB, Hoke A, Gill MJ, Power C (2010). Neurologic disease burden in treated HIV/AIDS predicts survival: a population-based study. Neurology.

[CR46] Wilkinson J, Radkowski M, Eschbacher JM, Laskus T (2010). Activation of brain macrophages/microglia cells in hepatitis C infection. Gut.

[CR47] Wilkinson J, Radkowski M, Laskus T (2009). Hepatitis C virus neuroinvasion: identification of infected cells. J Virol.

[CR48] Woods SP, Rippeth JD, Frol AB, Levy JK, Ryan E, Soukup VM, Hinkin CH, Lazzaretto D, Cherner M, Marcotte TD, Gelman BB, Morgello S, Singer EJ, Grant I, Heaton RK (2004). Interrater reliability of clinical ratings and neurocognitive diagnoses in HIV. J Clin Exp Neuropsychol.

